# Stress granules and RNA processing bodies are novel autoantibody targets in systemic sclerosis

**DOI:** 10.1186/s13075-016-0914-4

**Published:** 2016-01-22

**Authors:** Michael E. Johnson, Andrew V. Grassetti, Jaclyn N. Taroni, Shawn M. Lyons, Devin Schweppe, Jessica K. Gordon, Robert F. Spiera, Robert Lafyatis, Paul J. Anderson, Scott A. Gerber, Michael L. Whitfield

**Affiliations:** Department of Genetics, Geisel School of Medicine at Dartmouth, Hanover, NH USA; Division of Rheumatology, Immunology, and Allergy, Brigham and Women’s Hospital, Boston, MA USA; Department of Rheumatology, Hospital for Special Surgery, New York, NY USA; Boston University School of Medicine, Boston, MA USA; Dartmouth Medical School, Hinman Box 7400, Hanover, NH 03755 USA

**Keywords:** Systemic sclerosis, Scleroderma, Autoantibody, RNA processing bodies, Stress granules

## Abstract

**Background:**

Autoantibody profiles represent important patient stratification markers in systemic sclerosis (SSc). Here, we performed serum-immunoprecipitations with patient antibodies followed by mass spectrometry (LC-MS/MS) to obtain an unbiased view of all possible autoantibody targets and their associated molecular complexes recognized by SSc.

**Methods:**

HeLa whole cell lysates were immunoprecipitated (IP) using sera of patients with SSc clinically positive for autoantibodies against RNA polymerase III (RNAP3), topoisomerase 1 (TOP1), and centromere proteins (CENP). IP eluates were then analyzed by LC-MS/MS to identify novel proteins and complexes targeted in SSc. Target proteins were examined using a functional interaction network to identify major macromolecular complexes, with direct targets validated by IP-Western blots and immunofluorescence.

**Results:**

A wide range of peptides were detected across patients in each clinical autoantibody group. Each group contained peptides representing a broad spectrum of proteins in large macromolecular complexes, with significant overlap between groups. Network analyses revealed significant enrichment for proteins in RNA processing bodies (PB) and cytosolic stress granules (SG) across all SSc subtypes, which were confirmed by both Western blot and immunofluorescence.

**Conclusions:**

While strong reactivity was observed against major SSc autoantigens, such as RNAP3 and TOP1, there was overlap between groups with widespread reactivity seen against multiple proteins. Identification of PB and SG as major targets of the humoral immune response represents a novel SSc autoantigen and suggests a model in which a combination of chronic and acute cellular stresses result in aberrant cell death, leading to autoantibody generation directed against macromolecular nucleic acid-protein complexes.

**Electronic supplementary material:**

The online version of this article (doi:10.1186/s13075-016-0914-4) contains supplementary material, which is available to authorized users.

## Background

Systemic sclerosis (SSc) is a rare systemic autoimmune disease of unknown etiology characterized by skin fibrosis, internal organ involvement, vascular abnormalities, and autoantibody production. Patients are broadly classified as having either limited (lSSc) or diffuse (dSSc) disease based primarily upon the extent of skin involvement and autoantibody profiles. While a wide array of autoantibodies have been described for SSc, only a small number of these targets are used for clinical diagnosis and stratification. Autoantibodies targeting RNA polymerase III (RNAP3), topoisomerase 1 (TOP1; commonly referred to as Scl70), and centromere proteins (CENP) represent the three the most common, clinically measured autoantibodies observed in SSc [1, 2]. Other autoantibodies, including fibrillarin (U3RNP), Pm/Scl, Ku, U1RNP, U11/U12, and Th/To have also been described [1, 3] but are not routinely measured for clinical subtyping.

While the processes underlying autoantibody production in SSc remain poorly understood, the presence of certain autoantibodies is strongly predictive of clinical outcomes [1–3]. TOP1 and RNAP3 autoantibodies are almost exclusively seen in dSSc, while CENP, Th/To, and U1RNP antibodies are more commonly associated with lSSc [1, 3]. U3RNP autoantibodies are not associated with either clinical subset, and are often found in conjunction with other autoantibodies, including both TOP1 and CENP [3]. Certain antibodies, such as TOP1 and U11/12, have been shown to be predictive of poorer overall prognosis, including increased likelihood of pulmonary fibrosis [4] and cardiac involvement, while RNAP3 autoantibodies have recently been linked to co-occurrence of SSc with cancer [5].

Despite the importance of autoantibodies in SSc, the vast majority of target identification and phenotypic screening has been performed using methods targeting only a single autoantibody, with little ability to detect novel or low abundance autoantibodies. Furthermore, these methods fail to address the possibility of co-occurrence of multiple autoantibodies within a patient, which may have important clinical implications. Autoantigen microarrays have proven successful for screening large numbers of autoantibodies in parallel, however target identification is limited to those antigens produced and printed on the antigen microarrays [6]. To address these limitations, we performed immunoprecipitations (IP) of HeLa whole cell lysates using sera from RNAP3-, CENP-, and TOP1-positive patients, as well as healthy controls, followed by mass spectrometry (LC-MS/MS) to provide an unbiased assessment of all autoantibodies present in these SSc patients. This method provides a better view of the full range of autoantibodies present in SSc, including both novel and established targets, and provides insights into the general processes underlying autoantibody production.

## Methods

### Clinical samples

Patient serum was obtained from Boston University Medical School, Boston (BUMC), MA, USA and the Hospital for Special Surgery (HSS), New York, NY, USA. All relevant study protocols were approved by the Dartmouth College committee for the protection of human subjects, and the internal review boards of both BUMC and HSS. Informed consent was obtained from all patients prior to sample collection. Patients were diagnosed with either dSSc or limited SSc, as determined using the 1980 American College of Rheumatology classification criteria. Detection of major autoantibody reactivities was performed using standard clinical assays.

### Human cell lysates

HeLa cells were cultured in DMEM supplemented with 10 % fetal bovine serum (FBS) (v/v) and 100 IU/mL penicillin-streptomycin. Cells were grown to approximately 80 % confluence, harvested in IP lysis buffer (150 mM NaCl, 50 mM Tris pH 7.5, 1 mM MgCl_2_, 1 mM EDTA, 0.5 % Triton X-100, 2.5 mM β-mercaptoethanol, 1 mM sodium molybdate, 1 mM sodium fluoride, 1 mM sodium tartrate, 1 mM dithiothreitol (DTT), and protease inhibitors (Roche, Indianapolis, IN, USA)), lysed by passage through a pre-chilled high-gauge syringe, and centrifuged for 15 minutes to pellet debris. Lysates were then clarified by incubating for 4 h at 4 °C on a rotating platform. Protein concentrations were quantified using a standard bicinchoninic acid (BCA) protein assay kit (Thermo Scientific, Waltham, MA, USA).

### Serum immunoprecipitation

Patient serum was cross-linked to Protein G Dynabeads (Invitrogen, St. Louis, MO, USA) prior to IP. First, 100 μL serum (approximately 1 mg IgG) was added to 50 μL Protein G beads and incubated for 5 h at 4 °C. Samples were then washed in PBS, equilibrated in crosslinking buffer (50 mM HEPES, pH 8.2), and cross-linked to Protein G beads by the addition of 20 mM dimethyl pimelimidate and 300 mM HEPES (DMP) solution for 10 minutes at room temperature (repeated three times). The crosslinking reaction was then terminated by the addition of 50 mM ammonium bicarbonate, and the resulting antibody bead mixture added to 500 μL cell lysate (diluted to 4 mg/mL in IP lysis buffer). Samples were incubated overnight at 4 °C on a rotating platform, washed in cold IP lysis buffer, and eluted in a buffer containing 2 % SDS, 75 mM NaCl, 50 mM Tris pH 8.1, and 20 % glycerol at 65 °C for 5 minutes. Eluates were reduced by the addition of 0.1 M dithiothreitol (DTT) (to a final concentration 5 mM), and incubated at 80 °C for 5 minutes. Samples were then resolved by SDS-PAGE, split into high (>60 kDa) and low (<60 kDa) molecular weight fractions and analyzed by mass spectrometry.

### Mass spectrometry

Proteins contained in Coomassie-stained gel regions were digested overnight with trypsin (1:200 w/v) at 37 °C. Following digestion, peptides were extracted from the gels, dried, and analyzed by nanoscale LC-MS/MS. LC-MS/MS analyses were performed on either LTQ Orbitrap Classic or Orbitrap Fusion LC-MS/MS platforms. LTQ Orbitrap Classic analyses were conducted as described previously [7].

For Orbitrap Fusion analyses, samples were loaded onto an EASY-nLC 1000 Liquid Chromatograph (Thermo Scientific, Waltham, MA, USA) and separated by reverse-phase high pressure liquid chromatography (RP-HPLC) using an approximately 36-cm column with a 100-μM inner diameter packed with 3 μm 120 Å C_18_ particles (Dr. Maisch GmbH, Ammerbuch-Entringen, Germany). The resultant peptide eluate was directed into an Orbitrap Fusion Tribrid Mass Spectrometer operating in a data-dependent sequencing acquisition mode across a 30-minute reverse-phase gradient (6 % acetonitrile, 0.1 % formic acid to 30 % acetonitrile, 0.1 % formic acid) at 350 nL/min flow rate. The Orbitrap Fusion was operated with an Orbitrap MS1 scan at 120 K resolution, followed by Orbitrap MS2 scans of higher energy collision-induced dissociation (HCD) fragment ions (30 % HCD energy) at 15 K resolution using a maximum cycle type of 2 s, precursor ion dynamic exclusion window of 15 s, +2, +3, and +4 precursor ions selected for LC-MS/MS, and maximum ion injection times of 100 ms (MS1) and 50 ms (MS2). The resulting tandem mass spectra were data-searched using the COMET search engine [8] against a *Homo sapiens* proteome database (source: Uniprot; download date: 2 July 2013) with a precursor ion tolerance of +/- 1 Da [9] and a fragment ion tolerance of 0.02 Th. Peptide spectra matches (PSMs) were filtered to <1 % false discovery rate using the target decoy strategy [10], and reported.

### IP-western blots

Anti-UPF1 antibody was kindly provided by Dr. Lynne Maquat (University of Rochester Medical Center, Rochester, NY, USA). Antibodies to MOV10 and CAPRIN1 were purchased from Proteintech (Chicago, IL, USA); antibodies to G3BP1 and USP10 were purchased from Santa Cruz Biotechnology (Santa Cruz, CA, USA). Serum immunoprecipitation of HeLa lysates was performed as described above; 50 % of each eluate (15 μL) was then run on a 10 % bis-tris precast gel (Life Technologies, Carlsbad, CA, USA). HeLa whole cell lysate (100 μg) was used as a positive control; no loading control was performed due to the absence of viable targets present in all IP eluates. Western blots were then run following standard protocols, and visualized using Western Lightning ECL Pro or Ultra substrate (Perkin Elmer Inc., Waltham, MA, USA), as necessary.

### Data analysis

Non-redundant peptide hits, defined as mass spectra mapping exclusively to a given peptide fragment, were used for all downstream analyses. Pairwise comparisons between samples were performed by Fisher’s exact test using the Bonferroni correction for multiple hypothesis testing. Venn diagrams were generated using VENNY [11]. Network analysis was performed using the Genome-scale Integrated Analysis of gene Networks in Tissues (GIANT; http://giant.princeton.edu/) global network [12] and visualized using Cytoscape [13]. Communities in the network were detected using fast-greedy modularity as implemented in igraph. Functional annotation of individual communities was performed using g:Profiler [14]. Semiquantitative enrichment of SSc-associated autoantibodies was determined using a binary assessment of autoantibody presence or absence in a sample. Preferential enrichment in SSc was defined as all proteins detected in >50 % of all patient samples at a frequency >1.5-fold relative to controls. Enrichment of biological processes and cellular components was determined using g:Profiler using the g:SCS threshold correction for multiple hypothesis testing and a functional category size ≤500 genes. Hierarchical clustering was performed using Cluster 3.0 [15], and visualized using Java TreeView [16].

### Immunofluorescence

The day prior to the experiment, 10^5^ U2OS cells were seeded onto 11 mm glass coverslips and allowed to attach overnight at 37 °C/5 % CO_2_ in DMEM containing 10 % FBS (Gibco). Cells were treated with 100 μM sodium (meta)arsenite (Sigma Aldrich) for 1 h to induce the formation of stress granules and then with 4 % paraformaldehyde solution at room temperature for 15 minutes followed by blocking and permeabilization with 5 % normal horse serum, 0.1 % digitonin in Tris-buffered saline. Staining was performed with anti-eIF3b (Santa Cruz), anti-SK1-Hedls (Santa Cruz), and patient sera for 1 h at room temperature. Secondary antibodies (anti-goat-Cy3, anti-mouse-Cy2, and anti-human-Cy5) were purchased from Jackson Laboratories and incubated at room temperature for 1 h. Conventional fluorescence microscopy was performed using a microscope (model Elipse E800, Nikon, Tokyo, Japan) with epifluorescence optics with a digital camera (model CCD-SPOT RT; Diagnostic Instruments, Sterling Heights, MI). Images were compiled using Adobe Photoshop software (CS6; Adobe Systems, San Jose, CA).

## Results

### Identification of proteins cross-reacting to serum antibodies

Immunoprecipitations (IP) of HeLa whole cell lysates were performed using sera obtained from 13 SSc patients and 4 healthy controls. HeLa cells were chosen based upon their consistent, high level of expression of a broad range of proteins from the human genome [17].

SSc patients were divided into three groups, TOP1, RNAP3, and CENP, as measured in a reference laboratory; clinical data for each patient are shown in Table 1. These groups were chosen based upon their relative frequency, and their importance in clinical diagnosis. Immunoprecipitated proteins were analyzed by LC-MS/MS, and the resulting spectra aligned to the reference human proteome (UCSC version hg19). Data are presented in two ways; first to identify the total number of peptides that could be aligned to each protein (total hits), and second to identify all non-redundant peptides that mapped exclusively to a given protein (non-redundant hits). A complete list of all data can be found in Additional file 1: Table S1.

**Table 1:** Clinical information for patients involved in this study

Sample	Group	Age (years)	Sex	Race	Disease type	ILD/PAH	Disease duration (years)	ANA pattern	ANA titer	MRSS
SSc 1	TOP1	36	F	White	Diffuse	mild ILD	2.5		1:320	43
SSc 132	TOP1	49	F	White	Diffuse	No		Homogeneous	1:640	27
SSc 218	TOP1	55	F	White	Diffuse	ILD		Homogeneous/nucleolar	1:2560	18
SSc 208	TOP1	64	M	White	Diffuse	No		Nucleolar	1:1280	37
SSc 5	RNAP3	53	M	White	Diffuse	No	0.75	Speckled	1:80	36
SSc 7	RNAP3	45	F	Black	Diffuse	No	0.5	Speckled	1:80	27
SSc 10	RNAP3	52	M	White	Diffuse	No	0.5		0	22
SSc 18	RNAP3	69	F	White	Diffuse	ILD	0.5	Nucleolar	1:160	44
SSc 159	CENP	54	F	Mixed	Limited	No	7	Centromere	1:1280	2
SSc 177	CENP	64	F	White	Limited	No	15	Discrete speckled	4+	
SSc 194	CENP	66	F	White	Limited	No	18	Discrete speckled	4+	6
SSc 238	CENP	53	F	White	Limited	No	6	Centromere	1:640	5
SSc 226	CENP	55	F	Asian	Diffuse	No		Centromere	1:1280	6
HC 162	Control	24	M	White						
HC 400	Control	21	M	White						
HC 117	Control		M							
HC 118	Control		M							

Blank cells indicate information not available at the time of sample collection. *ILD* interstitial lung disease, *PAH* pulmonary arterial hypertension, *ANA* anti-nuclear antibody, *MRSS* modified Rodnan skin score, *SSc* systemic sclerosis, *M* male, *F* female

### Exclusivity and co-occurrence of SSc autoantibodies

**Fig. 1:**
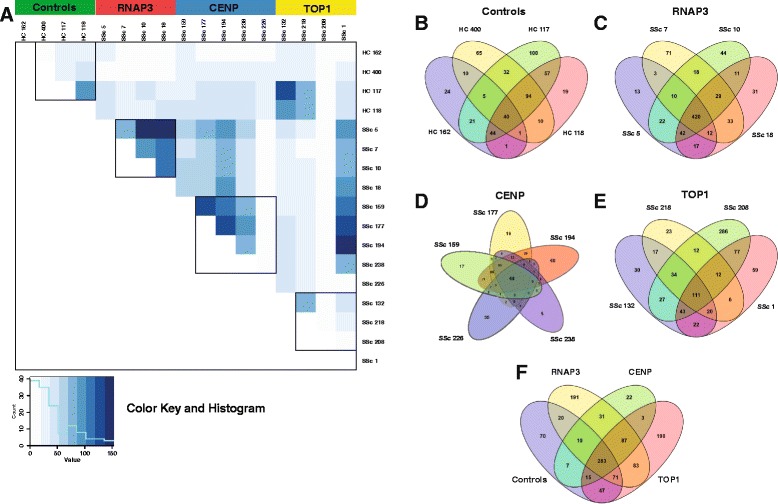
Overview of mass spectrometry results. **a** Correlation matrix of non-redundant protein hits for all patients and controls. Comparisons were performed using Fisher’s exact test with the Bonferroni correction. *Black boxes* indicate intra-group comparisons for each of the four clinically defined groups. *Green* controls; *red* RNA polymerase III (*RNAP3*); *blue* centromere protein (*CENP*); *yellow* topoisomerase I (*TOP1*). **b-f** *Venn diagrams* depict overlap in non-redundant peptide hits within and between groups. **b** healthy controls, **c** RNAP3, **d** CENP, **e** TOP1, and **f** overlap between groups

We observed a high degree of reproducibility between patients within their respective autoantibody groups (TOP1, RNAP3, and CENP; Fig. 1). The greatest degree of overlap between peptides was observed among RNAP3 patients (Fig. 1a and c), with 420 proteins (54.1 %) detected in all four patients (Fig. 1c). The remaining groups exhibited significant overlap in three of four (TOP1) and four of five (CENP) patients, respectively (Fig. 1a), along with a single outlier that showed either higher (SSc 208; TOP1) or lower (SSc 226; CENP) total peptide hits relative to other samples in these groups. Within TOP1, 111 proteins (14.2 %) were detected in all four patients (Fig. 1d), while CENP exhibited 48 proteins (10.5 %) common to all patients (Fig. 1c). The least overlap was seen in healthy controls, with only 40 proteins (7.6 %; Fig. 1b) common across individuals.

Across all samples, 283 proteins (25.0 %) were detected in at least one patient in each of the four autoantibody groups (Fig. 1e, Additional file 2: Table S2). Some of these proteins likely represent background signals (serum albumin (ALB), β-tubulin (TUBB), and ribosomal proteins), while others are considered specific to SSc despite trace level detection in controls. For example, multiple SSc autoantibody targets, including Ku (XRCC5 and XRCC6), Ro52/TRIM21, and nucleophosmin/B23 (NPM1) were present in this set of proteins. In contrast, 87 proteins (7.7 %) were detected in all three SSc groups, but were absent in controls (Fig. 1e; Additional file 2: Table S2). Functional analyses of these proteins revealed strong enrichment of proteins involved in oxidative stress responses and nucleic acid processing (Additional file 3: Table S3B).

Of the 1,130 non-redundant proteins identified, 473 (41.8 %) were unique to a given autoantibody group (Fig. 1f); however, the vast majority of these proteins were exclusive to a single patient, with only 111 (23.5 %) detected in two or more patients. These results suggest a wide range of autoantibody responses within each of the clinical autoantibody groups beyond what has already been described.

Among the major autoantibody groups, immunoprecipitation of RNAP3 was exclusive to the RNAP3 group, with no RNAP3 peptides detected in any of the other samples (Table 2). In contrast, TOP1 peptides were consistently highest among TOP1 patients, but were also detected at low levels in all four RNAP3^+^ patients, and in two controls (Table 2). As these patients were negative for TOP1 autoantibodies by clinical testing, these results indicate a higher degree of sensitivity for our IP/MS protocol compared to standard ELISA-based methods used clinically. In contrast, CENP was only detected at low levels in the CENP group, likely because it remained bound to the tightly packed centromere complex of chromatin.

**Table 2:** SSc-associated autoantibodies observed in this study

Alias	Associated proteins	Disease subset	Clinical associations	Prevalence in this dataset (avg/freq)				Reference
				Control (n = 4)	RNAP3 (n = 4)	TOP1 (n = 4)	CENP (n = 5)	
Major autoantibodies								
RNA Pol III	POLR3A	dSSc	Renal crisis, cancer	-	+++ (28/4)	-	-	Graf, et al. 2012 [1]; Mehra, et al. 2013 [3]
Scl70	TOP1	dSSc	Poor prognosis, internal organ involvement, and proteinuria	+ (3/2)	+ (4/4)	+++ (19/4)	-	Mehra, et al. 2013 [3]
Centromere	**CENPB**, CENPH	lSSc/CREST	PAH, ILD	-	-	-	+ (1/2)	Mehra, et al. 2013 [3]
Other SSc autoantibodies present in our dataset								
Endothelial Cell	TUBB, **VCL**, LMNA, RPLP0	SSc	PAH	+ (1/1)	++ (6/4)	+ (4/2)	+ (0/1)	Dib, et al. 2012 [34], Naniwa, et al. 2007 [35]
Fibroblast	ENO1, G6PD, **HSPA1A**, HSPA1B, VIM	SSc	PAH	+ (3/4)	+++ (12/4)	++ (5/3)	++ (8/5)	Terrier, et al. 2008 [36], Terrier, et al. 2009 [37]
Histone	H1FX, HIST1H1B, **HIST1H4A**	SSc	PF, internal organ involvement, decreased survival	+ (1/1)	+ (3/3)	+ (1/1)	-	Mehra, et al. 2013 [3]
B23	NPM1	dSSc, CENP- lSSc	PAH	+ (4/4)	++ (7/4)	++ (5/4)	++ (6/5)	Mehra, et al. 2013 [3]
Ku	**XRCC5**, XRCC6	lSSc	Myositis	+ (3/3)	+++ (12/4)	++ (8/4)	+ (2/3)	Graf, et al. 2012 [1]; Mehra, et al. 2013 [3]
Su	AGO2	SSc, PM/Scl	Unknown	-	+ (1/2)	+ (3/1)	-	Satoh, et al. 2013 [38]
Mitochondrial (M2)	**DLD**, PDHB	lSSc	Strong association with primary biliary cirrhosis	+ (1/1)	+ (2/3)	+ (1/1)	-	Mehra, et al. 2013 [13]
Pm/Scl	EXOSC1-**10**	SSc	PF, digital ulcers; decreased risk of PAH and GI symptoms	+ (2/2)	++ (5/3)	+ (2/2)	-	Mehra, et al. 2013 [13]
hnRNPs	**HNRNPA1**-3, HNRNPL	SSc	Common in SARDs	+ (0/1)	++ (7/4)	+ (3/4)	+ (2/4)	Siapka, et al. 2007 [39]
U1	**SNRNPA**, SPRNP70	SSc	Co-occurrence with SS-A/SS-B, PAH, overlap syndrome	-	+ (2/4)	+ (1/2)	+ (0/1)	Graf, et al. 2012 [1]; Mehra, et al. 2013 [3]
U5	SNRNP200	SSc, PM/Scl	Unknown	++ (6/3)	++ (9/4)	++ (8/3)	+ (1/2)	Kubo, et al. 2002 [40]
RO52/TRIM21	TRIM21	SSc	ILD, other autoimmune diseases	++ (6/3)	+++ (12/4)	++ (6/4)	++ (8/4)	Mehra, et al. 2013 [3]
RuvB	**RUVBL1**, RUVBL2	dSSc	Common in SARDs, older age at onset, male sex	+ (1/1)	++ (7/4)	+ (3/4)	+ (2/4)	Kaji, et al. 2014 [18]
Annexin V	ANXA5	dSSc, CENP- lSSc	Digital ischemia	+ (2/2)	++ (7/4)	+ (4/3)	+ (3/4)	Mehra, et al. 2013 [3]
SS-B/LA	SS-A, **SS-B**	SSc	ILD, other autoimmune diseases	-	+ (3/4)	+ (2/2)	+ (0/1)	Mehra, et al. 2013 [3]
Peroxiredoxin	PRDX1	SSc	Disease duration, PF, cardiac involvement, TOP1+ patients	+ (2/4)	++ (8/4)	+ (3/3)	+ (4/4)	Mehra, et al. 2013 [3]
hUBF/NOP90	UBTF	lSSc	Mild organ involvement, favorable prognosis	-	+ (1/2)	-	-	Mehra, et al. 2013 [3]
Th/To	POP1	lSSc	PF, renal crisis, poor prognosis, myositis, PAH	+ (1/2)	+ (1/1)	+ (3/3)	-	Graf, et al. 2012 [1]; Mehra, et al. 2013 [3]
PL-12	AARS	SSc, PM/DM	ILD without myositis	-	-	+ (1/1)	+ (1/1)	Hamaguchi, et al. 2013 [19]
OJ	IARS	SSc, PM/DM	ILD without myositis	+ (1/1)	-	+ (3/3)	-	Hamaguchi, et al. 2013 [19]
EJ	GARS	SSc, PM/DM	ILD, myositis	-	+ (3/4)	+ (2/2)	+ (0/1)	Hamaguchi, et al. 2013 [19]
Jo-1	HARS	SSc, PM/DM	ILD, myositis	-	+ (1/4)	-	-	Hamaguchi, et al. 2013 [19]
PL-7	TARS	SSc, PM/DM	ILD, myositis	+ (2/2)	++ (8/4)	++ (6/4)	+ (2/4)	Hamaguchi, et al. 2013 [19]
Ha	YARS	SSc, PM/DM	Interstitial pneumonia	-	+ (0/1)	-	-	Hashish, et al 2005 [41]
Zo	**FARSA**, FARSB	SSc, PM/Scl	Anti-synthetase syndrome	-	+ (2/4)	-	-	Betteridge, et al. 2007 [42]
SSc autoantibodies not detected in our dataset								
Fibrillarin	U3RNP	dSSc	More frequent in blacks; severe disease, poor prognosis	-	-	-	-	Mehra, et al. 2013 [3]
U11/U12 RNP	SNRNP35	SSc	Lung fibrosis, gastrointestinal involvement	-	-	-	-	Mimori, 1999 [43]
PDGFR	PDGFR	SSc	Unknown	-	-	-	-	Svegliati Baroni, et al. 2006 [44]
MMP	MMP family	dSSc	Skin, lung, and vascular fibrosis	-	-	-	-	Mehra, et al. 2013 [3]
tPA	PLAT	lSSc	PAH	-	-	-	-	Mehra, et al. 2013 [3]
IFI16	IFI16	lSSc	Common in SARDs	-	-	-	-	Mehra, et al. 2013 [3]
Fibrillin 1	FBN1	dSSc	Choctaw and Japanese patients; absent in Caucasians	-	-	-	-	Mehra, et al. 2013 [3]
Vascular Receptors	AGTR2, EDN1	SSc	TOP1+ patients, renal crisis	-	-	-	-	Mehra, et al. 2013 [3]
ATF2	ATF2	SSc	Longer disease duration, decreased lung function	-	-	-	-	Mehra, et al. 2013 [3]

Data are presented as the average of all peptide hits across each autoantibody group, followed by the frequency of peptide detection within the group. For autoantibodies known to target more than one protein or subunit, data for a single representative protein are shown, with the specific protein highlighted in bold. Associated proteins indicate specific protein targets identified in this study; among autoantibodies not identified here, the most common targets are listed. Symbols: -, +, ++, and +++ indicate an average of 0, 1–4, 5–9, and ≥10 peptide hits per group, respectively

*SSc* systemic sclerosis, *lSSc* limited cutaneous SSc, *dSSc* diffuse cutaneous SSc, *PAH* pulmonary arterial hypertension; *ILD* interstitial lung disease; *CREST* CREST syndrome (calcinosis, Raynaud phenomenon, esophageal dysmotility, sclerodactyly, and telangiectasia); *PM/Scl* polymyositis/scleroderma, *PM/DM* polymyositis/dermatomyositis

Other known SSc autoantigens were also detected. RuvBL [18] was strongly detected in all SSc samples, while virtually absent in controls. Ku and Su, along with a wide array of anti-tRNA synthetases [19] were routinely detected in both the RNAP3 and TOP1 subsets, but were only weakly present in the CENP and control groups (Table 2).

Several autoantigens previously implicated in SSc were found at low, background levels in both SSc and control samples. Ro52/TRIM21 [20] and nucleophosmin/B23 [21] were widely detected across all four groups, suggesting widespread reactivity to these proteins in SSc and controls. We did not find evidence in SSc of enrichment of Pm/Scl autoantibodies, which target exosome components EXOSC1-10 [22]. Peptides for these proteins were absent in the CENP group, but were detected at low levels in other subsets, including controls. Autoantigens not detected here include many of the URNPs, PDGFR, matrix metalloproteinases, tissue plasminogen activator, and vascular receptor antibodies (Table 2).

### Functional clustering of identified proteins

To identify functional interactions among autoantigens, all 763 non-redundant protein hits were submitted as a query to the GIANT global average network. This approach included both SSc-specific targets and those detected at background levels in controls, to better understand the full range of autoreactive proteins and complexes. Nine distinct communities were identified within the resulting network, in which each gene is represented by a node, and two genes share an edge if they are predicted to functionally interact (Additional file 4: Figure S1). Analysis of each of these communities by g:Profiler revealed functional enrichment for a wide range of biological processes associated with important disease processes and components (Additional file 4: Figure S1). Community 1 is dominated by ribosomal proteins, eukaryotic initiation factor 3 (eIF3) subunits, and includes the SSc autoantibody target nucleophosmin/B23. Communities 2 and 8 show strong enrichment for Gene Ontology (GO) terms mRNA processing, ribonucleoprotein complex, and cytosolic stress granule. Community 2 is dominated primarily by DEAD box helicases proteins, while community 8 contains a diverse array of proteins including multiple SSc autoantibodies, including TOP1, SSB, Pm/Scl proteins, URNPs, and HNRNPs, and numerous serine/arginine-rich splicing factors. Community 3 consists primarily of aminoacyl tRNA synthetases, a cluster often targeted in autoimmune diseases [19, 23]. Communities 4, 5, and 9 are strongly associated with a variety of GO processes known to play a major role in SSc, including wound healing, IFN signaling, and response to oxidative stress. Major proteins include CD44, HLAs, myosins, and filamin proteins in community 4 and tricarboxylic acid cycle proteins in community 5. Community 9 contains multiple protein disulfide isomerases and peroxiredoxins, protein folding enzymes such as calnexin (CANX) and calreticulin (CALR), and the major collagen processing enzyme prolyl 4-hydroxylase beta (P4HB). Community 6 contains multiple annexin and 14-3-3 proteins; enriched GO processes include ribonucleoprotein complex assembly, mitochondrial transport, RNA processing, and anchoring junction. Community 7 associated with GO terms include cell cycle, RNA polymerase III complex, DNA-PK-Ku complex, and antigen processing and presentation. Community 7 includes several SSc autoantibody targets including Ku proteins XRCC5 and 6, RUVBL1 and 2, RNA polymerase I and II subunits, multiple proteasomal subunits, and T-complex proteins.

### Preferential detection of autoantibodies in SSc

**Fig. 2:**
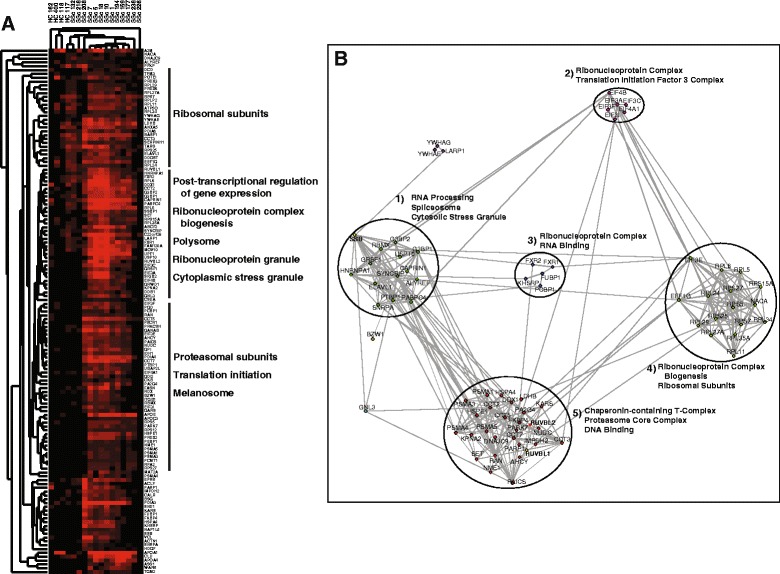
Proteins differentially detected in systemic sclerosis (*SSc*). Semiquantitative enrichment of SSc-associated autoantibodies was determined using a binary assessment of autoantibody presence or absence in a sample. Preferential enrichment in SSc was defined as all proteins detected in >50 % of all patient samples at a frequency >1.5-fold relative to controls. **a** Heat map of proteins differentially detected in SSc. **b** Network analysis of differentially detected proteins. Community detection was performed using the GIANT global network; functional annotation was performed using gProfiler

Subsequent comparisons between groups were performed in a semiquantitative manner based on the presence or absence of a given protein in an immunoprecipitant, with quantitative analyses limited to comparisons within an individual sample. To identify biological processes and cellular components differentially targeted in SSc, with minimal to no background detection in controls, we examined all proteins detected in >50 % of SSc samples at a frequency >1.5-fold relative to controls, resulting in a list of 137 differentially detected proteins (Fig. 2; Additional file 2: Table S2). Enriched biological processes included *ncRNA metabolic process*, response to oxygen radical, and triglyceride-rich lipoprotein particle remodeling. Preferentially targeted cellular components include cytosolic stress granule, lipid-protein complex*,* pigment granule, and anchoring junction; molecular functions include antioxidant activity and mRNA binding (Additional file 3: Table S3C).

### RNA processing centers are major targets of SSc autoantibodies

The strong enrichment for GO terms associated with mRNA processing and stress response, as well as the identification of cytosolic stress granule as an enriched cellular component, led us to further investigate the role of stress granules (SG) and RNA processing bodies (PB) in the autoantibody response of SSc. SG and PB represent distinct, non-membranous cytoplasmic entities, which arise in response to different cellular stresses including oxidative stress, hypoxia, viral infection, unfolded proteins, and amino acid deprivation [24]. These structures exist in constant flux, driven by the availability of constituent mRNPs, regulating the fate of untranslated mRNAs in response to translational arrest [25]. While SG are generally absent under normal conditions, PB are constitutively present at low levels due to their role as microRNA processing centers. Both structures have been shown to arise in response to cellular stresses, including oxidative stress, ischemia, and cancer [26], all of which are known to be important in SSc pathogenesis [5, 27].

In addition to the 137 differentially detected proteins described above, a wide range of PB/SG constituents were readily detected across most SSc samples (Additional file 5: Table S4). Substantial reactivity was seen against PB components such as UPF1 and MOV10, and SG proteins FXR1 and FXR2, G3BP1 and G3BP2, and USP10. Only background levels of reactivity were seen in healthy controls.

### Validation of PB/SG antibodies in SSc

**Fig. 3:**
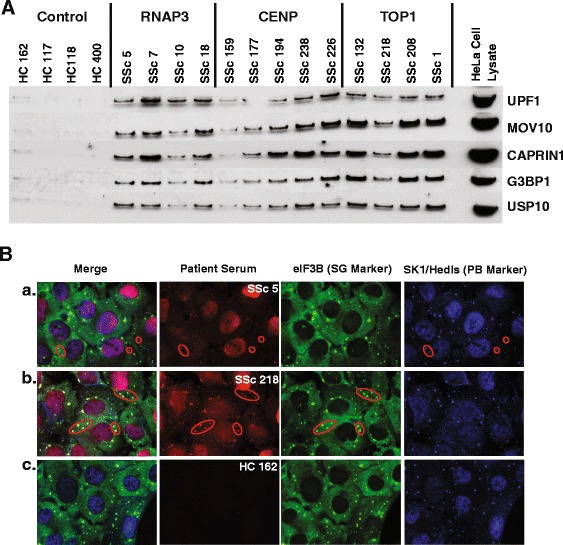
Validation of RNA processing bodies (*PB*)/stress granules (*SG*) as a target of the SSc autoimmune response. **a** HeLa cell lysates were immunoprecipitated using patient sera, resolved by SDS-PAGE, and probed with antibodies targeting known PB and SG proteins; HeLa whole cell lysate was used as a control. **b** Immunofluorescence was performed in U2OS cells treated with sodium (meta)arsenite to induce the formation of SG. Cells were then fixed with 4 % paraformaldehyde and permeabilized with 5 % normal horse serum and 0.1 % digitonin in Tris-buffered saline. Staining was performed with anti-eIF3b (SG marker), anti-SK1-Hedls (PB marker), and patient sera. Representative images depicting co-localization between patient sera and SG/PB markers are shown, with sites of co-localization circled in *red. RNAP* RNA polymerase, *CENP* centromere protein *TOPI* topoisomerase I

In order to validate the differential abundance of PB/SG proteins identified by LC-MS/MS, HeLa whole cell lysates were immunoprecipitated using antibodies from each patient as described in the LC-MS/MS analyses. Western blots were performed by resolving equal volumes of IP eluates by SDS-PAGE and transferring to nitrocellulose. Blots were then probed with antibodies targeting PB/SG proteins UPF1, MOV10, CAPRIN1, G3BP1, and USP10. These targets were chosen based upon their high level of detection across SSc groups, as determined based on our LC-MS/MS data. Strong reactivity was seen against all five proteins in SSc with only trace levels detected in controls (Fig. 3a), indicating widespread immune responses against these protein complexes.

Further validation was performed using immunofluorescence (IF) staining of U2OS cells maintained under conditions of oxidative stress to induce PB/SG formation. Cells were probed with patient sera in combination with PB and SG markers SK1-Hedls and eIF3b, respectively. Co-localization between patient sera and PB/SG markers was observed in six of nine SSc patients, with at least one positive sample in each of the three autoantibody groups; no staining was seen for any of the three healthy controls (Fig. 3b). These results are consistent with that seen on LC-MS/MS, particularly among RNAP3 patients, who exhibited the strongest and most consistent autoantibody responses across both methods. Taken together, these data strongly implicate PB/SG as novel targets of SSc autoantibody responses.

## Discussion

Autoantibodies have long been used in the diagnosis of SSc, with different autoantibodies predictive of clinical outcomes, including interstitial lung disease, pulmonary arterial hypertension, and skin involvement. While a wide array of SSc-associated autoantibodies have been described, diagnoses are often performed based upon the presence or absence of reactivity against three proteins: RNAP3, TOP1, and CENP. The data presented here suggest a much broader autoantibody response, which is reflective of underlying disease pathologies. Strong subset-specific reactivity was evident against both RNAP3 and TOP1, with no RNAP3 peptides detected in any of the other groups; however, all four RNAP3 patients exhibited modest reactivity against TOP1, indicating a degree of overlap between these two autoantibody groups. When peptides recovered are extended beyond the three major targets, we found substantial overlap across the three major SSc groups. We found peptides from the autoantigens of RuvBL1/2, which appear to act as general markers of SSc, with consistent detection across all SSc groups, with almost no reactivity seen in controls. In contrast, some common SSc autoantigens such as B23 and Ro52/TRIM21 were recovered in virtually all samples, and in controls, indicating an important degree of baseline reactivity against some of the more common autoantibody targets.

In this proof-of-concept study, we do not attempt to address the clinical implications of the autoantibody responses described here due to the limited number of patients analyzed, nor are we able to speculate on the presence or absence of these autoantibodies in other SSc autoantibody groups. Our depth in this study comes from the number of potential antigens analyzed, which cover the full proteome. Future studies examining a much larger cohort of SSc patients, along with representatives of other autoimmune diseases, will be necessary to determine the clinical value of these potential autoantibodies.

This is not the first study to suggest the presence of multiple autoantibodies in SSc. Immunoassays performed by Op De Beeck, et al. revealed the presence of multiple autoantibodies in a small subset of SSc patients [28]. A similar analysis by Graf et al. using the EUROLINE immunoassay revealed the presence of multiple autoantibodies in 11 % of patients [1].

Autoantibodies against extracellular immune signaling receptors and extracellular matrix proteins were conspicuously absent in these data; this includes the absence of numerous autoantibodies previously implicated in SSc pathogenesis, such as anti-fibrillin 1, anti-MMP, and anti-PDGFR [29]. Additional analyses in other cell types, such as fibroblasts or endothelial cells, and cells maintained under physiologically relevant growth conditions, such as immune activation or oxidative stress, may be useful for identifying other proteins and complexes which may play a role in disease pathogenesis.

In addition to identifying novel autoantibody targets, the unbiased nature of mass spectrometry provides additional insights into the processes potentially underlying autoimmunity. The preferential detection of proteins associated with RNA processing and oxidative stress as a general feature of SSc autoantibodies may be indicative of their origins. Combined with the consistent targeting of PB/SG described here spanning all SSc patients, these data suggest a basic model in which disease-specific pathologies give rise to specific autoantibodies. Strong induction of SGs is observed in response to cellular stresses, including oxidative stress and ischemia, two well-established phenomena in SSc [27]. SG/PB are also readily induced in response to the tumor microenvironment, consistent with recent evidence linking RNAP3-positive SSc and cancer [5, 30]. Combined with evidence linking transforming growth factor (TGF)-β signaling with an increase in PB formation [31], many of the major processes underlying SSc pathogenesis appear broadly consistent with an immune response against cells undergoing a stress response. PBs are also known to associate with other cytoplasmic structures, such as U bodies [32], which house an number of well-established SSc autoantibody targets, including U1, U5, and U11/U12. Taken together, these data suggest a model in which autoantibodies arise as a secondary phenotype in response to SSc-related processes already underway.

Some evidence suggesting a link between PBs and autoimmunity has been described previously. Bhanji, et al. observed reactivity against a number of PB-associated proteins, including GE1/*Hedls*, GW182, and Ago2 in a range of autoimmune diseases, including SSc [33]. Among these autoantigens, only Ago2 was also identified in this analysis, suggesting a persistent, yet diverse response against this complex. A similar analysis of SG-associated proteins has not been performed; however given the reactivity to PB proteins seen in other autoimmune diseases, reactivity in other autoimmune diseases is possible. A detailed analysis using both mass spectrometry and other methods will be necessary to understand the degree to which autoantibodies to SG/PBs are seen in other autoimmune diseases, and the clinical implications of these findings.

This work has several limitations. First, we cannot eliminate the possibility that some proteins found in our mass spectrometry data result from co-IP of multi-protein complexes by a single autoantibody; however, we were able to confirm the presence of multiple PB/SG autoantibodies by other means (Fig. 3). We also cannot rule out the possibility that some targets were missed due to their being sequestered into tightly packed molecular complexes associated with chromatin. For example, the presence of CENP autoantibodies within these samples had been established using clinical methods, indicating its absence in our mass spectrometry data is likely a result of its sequestration into large macromolecular complexes with limited solubility. A lack of age- and gender-matched controls likely underestimates the degree of baseline reactivity seen in unaffected controls. Finally, the small number of patient samples used in this study prevents any clinical interpretation, and the variability in the number of peptides recovered between experiments limits direct quantitative comparisons between autoantibody groups.

## Conclusions

The data presented here provide evidence of diverse immune reactivities in SSc targeting a wide array of protein complexes. Among these complexes, autoantibodies targeting PB/SG were consistently identified across both clinical SSc subsets and major autoantibody groups, suggesting a potential novel autoantibody target. Taken together, these data suggest immune responses to proteins involved in cellular stress may be a common mechanism for autoantibody generation.

## Additional files

Additional file 1: Table S1. Complete list of peptides identified in this analysis. *TP* number of total peptides mapping to a protein, *UP* number of unique peptides mapping to a protein, *UM* number of non-redundant peptides mapping exclusively to a protein, *MW* molecular weight, *Length* protein length in amino acids. (XLS 639 kb) Additional file 2: Table S2. Gene lists used in these analyses. (XLSX 17 kb) Additional file 3: Table S3. Systemic sclerosis (SSc)-specific enrichment of processes and components. Proteins differentially detected in SSc were analyzed using gProfiler. Statistically significant processes and components are shown. A) Peptides detected at any level across all four groups. B) Peptides identified in all SSc groups, but absent in controls. C) Analysis of 137 proteins differentially detected in SSc. *BP* biological process, *CC* cellular component, *MF* molecular function, *ke* KEGG pathway, *re* REACTOME pathway. (ODS 70 kb) Additional file 4: Figure S1. Network analysis of systemic sclerosis (SSc) autoantigens. All 763 non-redundant peptide hits identified in two or more patients were analyzed using the Genome-scale Integrated Analysis of gene Networks in Tissues (GIANT) global network to identify functionally associated protein networks. Analysis of community function was performed using gProfiler. SSc-associated autoantibodies are highlighted in *yellow*. (EPS 1902 kb) Additional file 5: Table S4. Processing body and stress granule proteins identified in this analysis. *Proteins with multiple subunits. Data indicate non-redundant peptide hits. (ODS 20 kb)

## Abbreviations

CENP: centromere protein
DMEM: Dulbecco’s modified Eagle’s medium
dSSc: diffuse systemic sclerosis
ELISA: enzyme-linked immunosorbent assay
FBS: fetal bovine serum
GIANT: Genome-scale Integrated Analysis of gene Networks in Tissues
IFN: interferon
kDa: kiloDalton
LC-MS/MS: liquid chromatography tandem-mass spectrometry
IP: immunoprecipitation
lSSc: limited systemic sclerosis
PB: RNA processing bodies
PBS: phosphate-buffered saline
RNAP3: RNA polymerase III
SG: stress granules
SSc: systemic sclerosis
TOP1: topoisomerase I

## References

[1] Graf SW, Hakendorf P, Lester S, Patterson K, Walker JG, Smith MD (2012). South Australian Scleroderma Register: autoantibodies as predictive biomarkers of phenotype and outcome. Int J Rheum Dis.

[2] Steen VD (2005). Autoantibodies in systemic sclerosis. Semin Arthritis Rheum..

[3] Mehra S, Walker J, Patterson K, Fritzler MJ (2013). Autoantibodies in systemic sclerosis. Autoimmun Rev.

[4] Fertig N, Domsic RT, Rodriguez-Reyna T, Kuwana M, Lucas M, Medsger TA (2009). Anti–U11/U12 RNP antibodies in systemic sclerosis: A new serologic marker associated with pulmonary fibrosis. Arthritis Care Res.

[5] Joseph CG, Darrah E, Shah AA, Skora AD, Casciola-Rosen LA, Wigley FM (2014). Association of the autoimmune disease scleroderma with an immunologic response to cancer. Science.

[6] Robinson WH, DiGennaro C, Hueber W, Haab BB, Kamachi M, Dean EJ (2002). Autoantigen microarrays for multiplex characterization of autoantibody responses. Nat Med.

[7] Yore MM, Kettenbach AN, Sporn MB, Gerber SA, Liby KT (2011). Proteomic analysis shows synthetic oleanane triterpenoid binds to mTOR. PLoS ONE.

[8] Eng JK, Jahan TA, Hoopmann MR (2013). Comet: An open-source MS/MS sequence database search tool. Proteomics.

[9] Hsieh EJ, Hoopmann MR, MacLean B, MacCoss MJ (2009). Comparison of database search strategies for high precursor mass accuracy MS/MS data. J Proteome Res.

[10] Elias JE, Gygi SP (2007). Target-decoy search strategy for increased confidence in large-scale protein identifications by mass spectrometry. Nat Methods.

[11] Oliveros JC. VENNY. An interactive tool for comparing lists with Venn Diagrams. 2007.

[12] Greene CS, Krishnan A, Wong AK, Ricciotti E, Zelaya RA, Himmelstein DS (2015). Zaslavsky E. Understanding multicellular function and disease with human tissue-specific networks. Nat Genet.

[13] Shannon P, Markiel A, Ozier O, Baliga NS, Wang JT, Ramage D (2003). Cytoscape: a software environment for integrated models of biomolecular interaction networks. Genome Res.

[14] Reimand J, Arak T, Vilo J (2011). g: Profiler—a web server for functional interpretation of gene lists (2011 update). Nucleic Acids Res.

[15] Eisen MB, Spellman PT, Brown PO, Botstein D (1998). Cluster analysis and display of genome-wide expression patterns. PNAS.

[16] Saldanha AJ (2004). Java Treeview—extensible visualization of microarray data. Bioinformatics.

[17] Novoradovskaya N, Perou C, Whitfield M, Basehore S, Pesich R, Aprelikova O, et al.: Universal human, mouse and rat reference RNA as standards for microarray experiments. In: Mol Bio Cell: 2002: Amer Soc Cell Biology 8120 Woodmont Ave, STE 750, Bethesda, MD 20814-2755 USA; 2002: 241A-241A.

[18] Kaji K, Fertig N, Medsger TA, Satoh T, Hoshino K, Hamaguchi Y (2014). Autoantibodies to RuvBL1 and RuvBL2: A novel systemic sclerosis-related antibody associated with diffuse cutaneous and skeletal muscle involvement. Arthr Care Res..

[19] Hamaguchi Y, Fujimoto M, Matsushita T, Kaji K, Komura K, Hasegawa M (2013). Common and Distinct Clinical Features in Adult Patients with Anti-Aminoacyl-tRNA Synthetase Antibodies: Heterogeneity within the Syndrome. PLoS ONE.

[20] Fujimoto M, Shimozuma M, Yazawa N, Kubo M, Ihn H, Sato S (1997). Prevalence and clinical relevance of 52-kDa and 60-kDa Ro/SS-A autoantibodies in Japanese patients with systemic sclerosis. Ann Rheum Dis.

[21] Ulanet DB, Wigley FM, Gelber AC, Rosen A (2003). Autoantibodies against B23, a nucleolar phosphoprotein, occur in scleroderma and are associated with pulmonary hypertension. Arth Care Res.

[22] Brouwer R, Vree Egberts WTM, Hengstman GJD, Raijmakers R, van Engelen BGM, Peter Seelig H (2002). Autoantibodies directed to novel components of the PM/Scl complex, the human exosome. Arth Res.

[23] Lega J-C, Fabien N, Reynaud Q, Durieu I, Durupt S, Dutertre M (2014). The clinical phenotype associated with myositis-specific and associated autoantibodies: A meta-analysis revisiting the so-called antisynthetase syndrome. Autoimmun Rev.

[24] Kedersha N, Ivanov P, Anderson P (2013). Stress granules and cell signaling: more than just a passing phase?. Trends Biochem Sci.

[25] Kedersha N, Anderson P (2009). Regulation of translation by stress granules and processing bodies. Prog Mol Biol Transl Sci..

[26] Anderson P, Kedersha N (2008). Stress granules: the Tao of RNA triage. Trends Biochem Sci.

[27] Katsumoto TR, Whitfield ML, Connolly MK (2011). The pathogenesis of systemic sclerosis. Annu Rev Pathol-Mech..

[28] Op De Beéck K, Vermeersch P, Verschueren P, Westhovens R, Mariën G, Blockmans D (2012). Antinuclear antibody detection by automated multiplex immunoassay in untreated patients at the time of diagnosis. Autoimmun Rev.

[29] Chung L, Utz P (2004). Antibodies in scleroderma: Direct pathogenicity and phenotypic associations. Curr Rheumatol Rep.

[30] Anderson P, Kedersha N, Ivanov P. Stress granules, P-bodies and cancer. Biochim Biophys Acta. 2015, 1849, 861–870. 10.1016/j.bbagrm.2014.11.009 PMC4457708 25482014

[31] Blanco FF, Sanduja S, Deane NG, Blackshear PJ, Dixon DA (2014). Transforming growth factor β regulates P-body formation through induction of the mRNA decay factor tristetraprolin. Mol Cell Bio.

[32] Liu J-L, Gall JG (2007). U bodies are cytoplasmic structures that contain uridine-rich small nuclear ribonucleoproteins and associate with P bodies. PNAS.

[33] Bhanji RA, Eystathioy T, Chan EKL, Bloch DB, Fritzler MJ (2007). Clinical and serological features of patients with autoantibodies to GW/P bodies. Clin Immunol.

[34] Dib H, Tamby MC, Bussone G, Regent A, Berezné A, Lafine C, Broussard C, Simonneau G, Guillevin L, Witko-Sarsat V (2012). Targets of anti-endothelial cell antibodies in pulmonary hypertension and scleroderma. Eur Respir J.

[35] Naniwa T, Sugiura Y, Banno S, Yoshinouchi T, Matsumoto Y, Ueda R (2007). Ribosomal P protein P0 as a candidate for the target antigen of anti-endothelial cell antibodies in mixed connective tissue. Clin Exp Rheumatol..

[36] Terrier B, Tamby MC, Camoin L, Guilpain P, Broussard C, Bussone G (2008). Identification of target antigens of antifibroblast antibodies in pulmonary arterial hypertension. Am J Resp Crit Care Med.

[37] Terrier B, Tamby MC, Camoin L, Guilpain P, Bérézné A, Tamas N, et al. Anti-fibroblast antibodies from systemic sclerosis patients bind to α-enolase and are associated with interstitial lung disease. Ann Rheum Dis. 2009. 10.1136/ard.2008.104299 19293162

[38] Satoh M, Chan JY, Ceribelli A, del-Mercado MV, Chan EK. Autoantibodies to Argonaute 2 (Su antigen). In: Ten Years of Progress in GW/P Body Research. Springer. 2013:45-59. 10.1007/978-1-4614-5107-5_4 23224964

[39] Siapka S, Patrinou-Georgoula M, Vlachoyiannopoulos PG, Guialis A (2007). Multiple specificities of autoantibodies against hnRNP A/B proteins in systemic rheumatic diseases and hnRNP L as an associated novel autoantigen. Autoimmunity.

[40] Kubo M, Ihn H, Kuwana M, Asano Y, Tamaki T, Yamane K, Tamaki K (2002). Anti-U5 snRNP antibody as a possible serological marker for scleroderma–polymyositis overlap. Rheumatol.

[41] Hashish L, Trieu E, Sadanandan P, Targoff I. Identification of autoantibodies to tyrosyl-tRNA synthetase in dermatomyositis with features consistent with anti-synthetase syndrome. In: Arthritis Rheum. 2005; 2005: S312-S312

[42] Betteridge Z, Gunawardena H, North J, Slinn J, McHugh N (2007). Anti-synthetase syndrome: a new autoantibody to phenylalanyl transfer RNA synthetase (anti-Zo) associated with polymyositis and interstitial pneumonia. Rheumatol.

[43] Mimori T (1999). Autoantibodies in Connective Tissue Diseases. Clinical Significance and Analysis of Target Autoantigens. Internal Med.

[44] Svegliati BS, Santillo M, Bevilacqua F, Luchetti M, Spadoni T, Mancini M (2006). Stimulatory autoantibodies to the PDGF receptor in systemic sclerosis. N Engl J Med.

